# Supplementary material for: Exercise versus Metformin to Improve Pregnancy Outcomes among Overweight Pregnant Women: A Systematic Review and Network Meta-Analysis
Source: J Clin Med. 2021 Aug 7;10(16):3490. doi: 10.3390/jcm10163490 (PMC8397096; doi:10.3390/jcm10163490)

Table S1. Direct estimates obtained.

Reference	Intervention	GDM		HDP		MWG	
		RR	95% CI	RR	95% CI	$\Delta\bar{x}$	95% CI
Kong K et al (2014)-1	Aerobic	1.00	(0.07, 13.64)	1.00	(0.02, 45.63)	0.59	(-4.74, 5.92)
Kong K et al (2014)-2	Aerobic	1.10	(0.02, 50.43)	3.30	(0.15, 72.08)	- 0.41	(-8.31, 7.49)
Seneviratne SN et al (2016)	Aerobic	2.00	(0.39, 10.26)	2.00	(0.19, 21.11)	- 1.20	(-3.73, 1.33)
Wang C et al (2017)	Aerobic	0.54	(0.37, 0.79)	0.88	(0.50, 1.53)	- 2.09	(-2.99, -1.19)
Barakat R et al (2016)-1	Combined	0.43	(0.08, 2.30)	0.58	(0.17, 1.97)	-	-
Barakat R et al (2016)-2	Combined	1.16	(0.08, 17.60)	2.32	(0.22, 24.09)	-	-
Bisson M et al (2015)	Combined	0.60	(0.16, 2.23)	0.67	(0.12, 3.64)	0.10	(-2.74, 2.94)
Daly N et al (2017)	Combined	1.19	(0.80, 1.77)	-	-	- 1.70	(-4.19, 0.79)
Garnæs KK et al (2016)	Combined	0.59	(0.21, 1.64)	0.41	(0.11, 1.45)	1.30	(-1.50, 4.10)
Nascimento SL et al (2011)	Combined	-	-	-	-	- 1.20	(-3.96, 1.56)
Oostdam N et al (2012)	Combined	0.68	(0.28, 1.60)	-	-	0.60	(-1.24, 2.44)
Ruiz JR et al (2013)	Combined	0.66	(0.29, 1.52)	0.71	(0.29, 1.74)	- 0.50	(-1.52, 0.52)
Barakat R et al (2009) – 1	Resistance	-	-	-	-	- 1.40	(-4.67, 1.87)
Barakat R et al (2009) – 2	Resistance	-	-	-	-	- 1.30	(-4.34, 1.74)
Abd El Fattah EA et al (2016)	Metformin	0.33	(0.07, 1.61)	0.33	(0.07, 1.61)	- 5.06	(-5.82, -4.30)
Brink HS et al (2018)	Metformin	0.65	(0.37, 1.14)	0.21	(0.01, 4.12)	-	-
Chiswick C et al (2015)	Metformin	0.78	(0.50, 1.22)	1.65	(0.93, 2.93)	- 0.53	(-1.78, 0.72)
Nascimento IB et al (2020)	Metformin	0.67	(0.31, 1.45)	0.18	(0.08, 0.42)	-	-
Syngelaki A et al (2016)	Metformin	1.10	(0.64, 1.88)	0.52	(0.31, 0.88)	-	-

Table S2. Excluded trials with reasons.

Reference	Intervention	Main reason for exclusion
Brislane Á et al (2021) [79]	Aerobic exercise	Total or normalweight women
Calvalcante SR et al (2009) [80]	Aerobic exercise	Total or normalweight women
Clapp JF et al (2000) [81]	Aerobic exercise	Total or normalweight women
de Oliveria Melo AS et al (2012) [82]	Aerobic exercise	Total or normalweight women
Ghodsi Z et al (2014) [83]	Aerobic exercise	Total or normalweight women
Guelfi KJ et al (2016) [84]	Aerobic exercise	Total or normalweight women
Hopkins SA et al (2011) [85]	Aerobic exercise	Total or normalweight women
Kasawara KT et al (2013) [86]	Aerobic exercise	Other risk factors
Khoram S et al (2019) [87]	Aerobic exercise	Other risk factors
Kihlstrand M et al (1999) [88]	Aerobic exercise	Total or normalweight women
Ko CW et al (2014) [89]	Aerobic exercise	Total or normalweight women
Labonte-Lemoyne E et al (2017) [90]	Aerobic exercise	Total or normalweight women
Marquez-Sterling S et al (2000) [91]	Aerobic exercise	Total or normalweight women
McDonald SM et al (2020) [92]	Aerobic exercise	Total or normalweight women
Navas A et al (2021) [93]	Aerobic exercise	Total or normalweight women
Sedaghati P et al (2007) [94]	Aerobic exercise	Total or normalweight women
Taniguchi C et al (2016) [95]	Aerobic exercise	Total or normalweight women
Tomic V et al (2013) [96]	Aerobic exercise	Total or normalweight women
Wen-Chun S et al (2021) [97]	Aerobic exercise	Total or normalweight women
Rakhshani A et al (2012) [98]	Alternative exercise	Total or normalweight women
Sonmezer E et al (2021) [99]	Alternative exercise	Total or normalweight women
Sun YC et al (2010) [100]	Alternative exercise	Total or normalweight women
Bacchi M et al (2018) [101]	Combined exercise	Total or normalweight women
Backhausen MG et al (2017) [102]	Combined exercise	Total or normalweight women
Barakat R et al (2011) [103]	Combined exercise	Total or normalweight women
Barakat R et al (2012) [104]	Combined exercise	Total or normalweight women
Barakat R et al (2013) [105]	Combined exercise	Total or normalweight women
Barakat R et al (2018) [106]	Combined exercise	Total or normalweight women
Barakat R et al (2019) [107]	Combined exercise	Total or normalweight women
Brik M et al (2018) [108]	Combined exercise	Total or normalweight women
Broberg L et al (2021) [109]	Combined exercise	Total or normalweight women
Cordero Y et al (2012) [110]	Combined exercise	Total or normalweight women
Cordero Y et al (2015) [111]	Combined exercise	Total or normalweight women
da Silva SG et al (2017) [112]	Combined exercise	Total or normalweight women
Fernández-Buhigas I et al (2020) [113]	Combined exercise	Total or normalweight women
Haakstad LA et al (2011) [114]	Combined exercise	Total or normalweight women
Peláez M et al (2019) [115]	Combined exercise	Total or normalweight women
Perales M et al (2016) [116]	Combined exercise	Total or normalweight women
Price BB et al (2012) [117]	Combined exercise	Total or normalweight women
Ramírez-Vélez R et al (2017) [118]	Combined exercise	Total or normalweight women
Rodríguez-Blanque R et al (2017) [119]	Combined exercise	Total or normalweight women
Stafne SN et al (2012) [120]	Combined exercise	Total or normalweight women
Abd El Hameed AA et al (2011) [121]	Metformin	PCOS women
Begum MR et al (2009) [122]	Metformin	PCOS women
Glueck CJ et al (2002) [123]	Metformin	PCOS women
Jamal A et al (2012) [124]	Metformin	PCOS women
Khattab S et al (2011) [125]	Metformin	PCOS women
Løvvik TS et al (2019) [126]	Metformin	PCOS women
Valdés E et al (2018) [127]	Metformin	Other risk factors
Vanky E et al (2004) [128]	Metformin	PCOS women
Vanky E et al (2010) [129]	Metformin	PCOS women

Table S3. Characteristics of the interventions.

Reference	Intervention	Control
**A. Exercise**		
Barakat R et al (2009) [54]	Duration: 26 weeks (12-13 wk until 38-39 wk).<br>Frequency: 3 times/week.<br>Exercises: 35-40 min. Warm up/cool-down, toning and joint mobilization exercises, barbells (3kg), low-medium resistance bands.<br>Type: Resistance exercise.<br>Intensity: Light-Moderate (<80% of predicted maximum heart rate).	The women were asked to maintain their physical activity during the study period.
Barakat R et al (2016) [47]	Duration: 28 weeks (9-11 wk until 38-39 wk).<br>Frequency: 3 times/week.<br>Exercises: 50-55 min. Warm up/cool-down, walking, aerobic exercise, aerobic dance, resistance exercises (2 kg weights, low resistance bands), flexibility, relaxation stretching, PFMT.<br>Type: Combined exercise.<br>Intensity: Moderate (<70% of predicted maximun heart rate, Borg: 12-14).	The women received general advice from their healthcare provider about the positive effects of physical activity.<br>They had the same visits with health care providers as the intervention group.<br>A questionnaire was conducted once a quarter. Those women who exercised continuously were excluded from the analysis.
Bisson M et al (2015) [48]	Duration: 12 weeks (15 wk until 27 wk).<br>Frequency: 3 times/week.<br>Exercises: 60 min. Warm up/cool-down, stationary ergocycle, treadmill walk, muscular work-out.<br>Type: Combined exercise.<br>Intensity: Moderate (70% of peak heart rate, modified Borg: 3-5).	The women were asked to continue with their usual activities, without restricting physical activity.<br>Both groups received a pamphlet of the benefits of physical activity and the exercises they could do.
Daly N et al (2017) [49]	Duration: 22 weeks (>17 wk until delivery).<br>Frequency: 3 times week.<br>Exercises: 50-60 min. Warm up/cool-down, resistance or weight exercises, aerobic exercises, PFMT.<br>Type: Combined exercise.<br>Intensity: NA.	The women received written information about the exercise.<br>Both groups received a pamphlet with information about healthy eating.
Garnæs KK et al (2016) [50]	Duration: 25 weeks (12-18 wk until 39 wk).<br>Frequency: 4 times/week.<br>Exercises: 60 min. Walking/jogging, resistance training, PFMT.<br>Type: Combined exercise.<br>Intensity: Moderate (~80% of maximal capacity, Borg: 12-15).	Information on healthy eating and lifestyles was provided.<br>The pregnant women were asked to continue with their usual activities and were not discouraged from exercising.
Kong K et al (2014) [44]	Duration: 20 weeks (15 wk until 35 wk).<br>Frequency: 5 times/week.<br>Exercises: 30 min. Walk (Treadmills).<br>Type: Aerobic exercise.<br>Intensity: Moderate (cadence ≥80 steps per minute).	The women received no recommendations for physical activity, but they were not discouraged from doing so.
Nascimento SL et al (2011) [51]	Duration: ~18 weeks (~20 wk until delivery).<br>Frequency: 5 times/week.	The women received no recommendations for physical activity.

	Exercises: 40 min. General stretching, strength exercises, relaxation, walking.<br>Type: Combined exercise.<br>Intensity: Moderate (<140 bpm of HR).	Both groups received standardized nutritional counseling.
Oostdam N et al (2012) [52]	Duration: 24 weeks (15 wk until 39 wk).<br>Frequency: 2 times/week.<br>Exercises: 60 min. Warm up/cool-down, aerobic and strength exercises.<br>Type: Combined exercise.<br>Intensity: Moderate (Borg: 12).	The women did not receive any exercise program, but received the usual care, which is the same as women of a healthy weight.
Ruiz JR et al (2013) [53]	Duration: 30 weeks (9 wk until 39 wk).<br>Frequency: 3 times week.<br>Exercises: 50-55 min. Warm-up/cool-down, waking, stretching, aerobic exercises, aerobic dance, resistance exercises, barbells (3kg), low-medium elastic bands.<br>Type: Combined exercise.<br>Intensity: Light-Moderate (<60% age predicted maximum heart rate, Borg: 10-12).	The women received standard care. Counseling on nutrition and physical activity was included, and exercise was not discouraged.
Seneviratne SN et al (2016) [45]	Duration: 15 weeks (20 wk until 35 wk).<br>Frequency: 3-5 times/week.<br>Exercises: 15-30 min. Stationary bicycle.<br>Type: Aerobic exercise.<br>Intensity: Moderate (40–59% VO_2 reserve).	The women did not receive the exercise program.
Wang C et al (2017) [46]	Duration: 25 weeks (11 wk until 36 wk).<br>Frequency: 3 times/week.<br>Exercises: 45-60 min. Interval stationary cycling (Intensity intervals between moderate level, 65-75% of the age-predicted HRmax, Borg 12-14, and vigorous level, 75-85% of the age-predicted maximum heart rate, Borg 15-16).<br>Type: Aerobic exercise.<br>Intensity: Moderate-Vigorous.	The women continued with their usual activities, and physical exercise was not discouraged.
**B. Metformin**		
Abd El Fattah EA et al (2016) [55]	Duration: 20 weeks (early 2nd trimester until 36 wk).<br>Dose: 1000 mg/day.	Placebo was administered.
Brink HS et al (2018) [56]	Duration: 25 weeks (14 wk until 39 wk).<br>Dose: 1000 mg/day.	Women in both groups followed a 2000 Kcal/day diet.
Chiswick C et al (2015) [57]	Duration: 25 weeks (12-16 wk until 39 wk).<br>Dose: 2500 mg/day.	Placebo was administered.
Nascimento IB et al (2020) [58]	Duration: >20 weeks (<20 wk until delivery).<br>Dose: 1000 mg/day.	Women in both groups received the usual health care.<br>Both groups received nutritional and physical activity recommendations.
Syngelaki A et al (2016) [59]	Duration: 25 weeks (12-18 wk until delivery).<br>Dose: 3000 mg/day.	Placebo was administered.<br>Both groups received nutritional and physical activity recommendations.

PFMT: pelvic floor muscle training; NA: Not available

Table S4. Measure of outcomes.

Reference	Gestational diabetes mellitus	Hypertensive disorders
Barakat R et al (2009) [54]	Not applicable	Not applicable
Barakat R et al (2016) [47]	Medical records	Gestational hypertension
Bisson M et al (2015) [48]	Not available	Gestational hypertension
Daly N et al (2017) [49]	75-g oral glucose tolerance test (International Association of Diabetes in Pregnancy Studies Group criteria)	Not applicable
Garnæs KK et al (2016) [50]	75-g oral glucose tolerance test (WHO/IADPSG 2013 definition)	Gestational hypertension
Kong K et al (2014) [44]	Not available	Gestational hypertension + preeclampsia
Nascimento SL et al (2011) [51]	Not applicable	Not applicable
Oostdam N et al (2012) [52]	Not available	Not applicable
Ruiz JR et al (2013) [53]	Not available	Gestational hypertension
Seneviratne SN et al (2016) [45]	75-g oral glucose tolerance test	Gestational hypertension + Preeclampsia
Wang C et al (2017) [46]	75-g oral glucose tolerance test	Gestational hypertension + Preeclampsia
Abd El Fattah EA et al (2016) [55]	75-g oral glucose tolerance test	Gestational hypertension
Brink HS et al (2018) [56]	75-g oral glucose tolerance test	Preeclampsia
Chiswick C et al (2015) [57]	75-g oral glucose tolerance test	Gestational hypertension + Preeclampsia
Nascimento IB et al (2020) [58]	World Health Organization criteria	Preeclampsia
Syngelaki A et al (2016) [59]	75-g oral glucose tolerance test	Gestational hypertension + Preeclampsia

Not applicable: if not measure gestational diabetes mellitus; Not available: if there is not information.

Table S5. Grades of Recommendation, Assessment, Development, and Evaluation.

Certainty assessment							Impact	Certainty
№ of studies	Study design	Risk of bias	Inconsistency	Indirectness	Imprecision	Other considerations		
A. Gestational diabetes mellitus								
Aerobic exercise								
3	RCT	very serious	serious	serious	not serious	very strong association	Meta-analysis: RR = 0.59 (0.41, 0.85)<br>NMA: RR = 0.51 (0.26, 0.97)	LOW
Combined exercise								
6	RCT	serious	serious	serious	not serious	none	Meta-analysis: RR = 0.91 (0.67, 1.22)<br>NMA: RR = 0.75 (0.47, 1.19)	VERY LOW
Metformin								
5	RCT	very serious	serious	serious	not serious	none	Meta-analysis: RR = 0.78 [0.59, 1.02]<br>NMA: RR = 0.74 (0.49, 1.10)	VERY LOW
B. Hypertensive disorders of pregnancy								
Aerobic exercise								
3	RCT	very serious	serious	serious	serious	none	Meta-analysis: RR = 0.95 (0.56, 1.62)<br>NMA: RR = 1.19 (0.36, 3.89)	VERY LOW
Combined exercise								
4	RCT	serious	serious	serious	not serious	strong association	Meta-analysis: RR = 0.65 (0.36, 1.14)<br>NMA. RR = 0.64 (0.26, 1.57)	LOW
Metformin								
5	RCT	very serious	serious	serious	not serious	strong association	Meta-analysis: RR = 0.48 (0.19, 1.22)<br>NMA: RR = 0.47 (0.22, 1.04)	VERY LOW
C. Maternal weight gain								
Aerobic exercise								
3	RCT	very serious	serious	serious	not serious	publication bias strongly suspected very strong association	Meta-analysis: MWG = -1.91 Kg (-2.74, -1.07)<br>NMA: MWG = -1.40 Kg (-3.47, 0.68)	VERY LOW
Combined exercise								
6	RCT	serious	serious	serious	not serious	publication bias strongly suspected	Meta-analysis: MWG = -0.31 Kg (-1.06, 0.44)<br>NMA: MWG = -0.24 Kg (-1.68, 1.20)	VERY LOW
Resistance Exercise								
1	RCT	not serious	not serious	serious	serious	publication bias strongly suspected strong association	Meta-analysis: MWG = -1.35 Kg (-3.57, 0.88)<br>NMA. MWG = -1.35 Kg (-4.29, 1.59)	LOW
Metformin								
2	RCT	very serious	serious	serious	serious	publication bias strongly suspected very strong association	Meta-analysis: MWG = -2.82 Kg (-7.26, 1.62)<br>NMA: MWG = -2.93 (-4.98, -0.87)	VERY LOW

Table S6. Transitivity analysis.

A. Gestational diabetes mellitus

**Reference**	**Age**				**BMI**			
	**Intervention**		**Control**		**Intervention**		**Control**	
	**x̄**	**SD**	**x̄**	**SD**	**x̄**	**SD**	**x̄**	**SD**
**Physical exercise**								
Barakat R et al (2016)-1	-	-	-	-	-	-	-	-
Barakat R et al (2016)-2	-	-	-	-	-	-	-	-
Bisson M et al (2015)	30.5	3.7	31.0	4.0	34.6	5.4	33.9	4.5
Daly N et al (2017)	30.0	5.1	29.4	4.8	34.7	4.6	34.7	5.1
Garnæs KK et al (2016)	31.3	3.8	31.4	4.7	33.9	3.8	35.1	4.6
Kong K et al (2014)-1	26.2	2.6	27.3	3.6	26.5	1.2	27.4	1.4
Kong K et al (2014)-2	28.6	5.3	25.7	4.0	34.7	4.6	34.2	3.6
Oostdam N et al (2012)	30.8	5.2	30.1	4.5	33.0	3.7	33.9	5.6
Ruiz JR et al (2013)	-	-	-	-	-	-	-	-
Seneviratne SN et al (2016)	-	-	-	-	32.1	4.4	34.1	5.9
Wang C et al (2017)	32.1	4.6	32.5	4.9	26.8	2.7	26.8	2.8
**Pooled**	**30.1**	**1.9**	**29.8**	**2.1**	**31.9***	**3.9**	**32.5***	**4.2**
**Metformin**								
Abd El Fattah EA et al (2016)	26.9	5.2	26.2	5.5	36.4	1.0	36.2	0.9
Brink HS et al (2018)	29.3	5.2	30.7	5.2	31.3	5.8	30.0	5.5
Chiswick C et al (2015)	28.7	5.8	28.9	5.1	37.8	4.7	37.5	5.5
Nascimento IB et al (2020)	28.6	6.2	29.6	6.1	37.5	4.6	37.2	5.7
Syngelaki A et al (2016)	-	-	-	-	-	-	-	-
**Pooled**	**28.3**	**1.0**	**28.8**	**1.7**	**36.4***	**1.2**	**35.7***	**1.5**

From the confidence intervals of the pooled (not shown), the standard error was obtained. And taking as N the number of valid studies included in each pooled, the standard deviation of each pooled was calculated. *Statistically significant differences

B. Hypertensive disorders of pregnancy

**Reference**	**Age**				**BMI**			
	**Intervention**		**Control**		**Intervention**		**Control**	
	**x̄**	**SD**	**x̄**	**SD**	**x̄**	**SD**	**x̄**	**SD**
**Physical exercise**								
Barakat R et al (2016)-1	-	-	-	-	-	-	-	-
Barakat R et al (2016)-2	-	-	-	-	-	-	-	-
Bisson M et al (2015)	30.5	3.7	31.0	4.0	34.6	5.4	33.9	4.5
Garnæs KK et al (2016)	31.3	3.8	31.4	4.7	33.9	3.8	35.1	4.6
Kong K et al (2014)-1	26.2	2.6	27.3	3.6	26.5	1.2	27.4	1.4
Kong K et al (2014)-2	28.6	5.3	25.7	4.0	34.7	4.6	34.2	3.6
Ruiz JR et al (2013)	-	-	-	-	-	-	-	-
Seneviratne SN et al (2016)	-	-	-	-	32.1	4.4	34.1	5.9
Wang C et al (2017)	32.1	4.6	32.5	4.9	26.8	2.7	26.8	2.8
**Pooled**	**29.9**	**2.3**	**29.8**	**2.5**	**31.3***	**3.6**	**31.8***	**3.9**
**Metformin**								
Abd El Fattah EA et al (2016)	26.9	5.2	26.2	5.5	36.4	1.04	36.2	0.99
Brink HS et al (2018)	29.3	5.2	30.7	5.2	31.3	5.8	30	5.5
Chiswick C et al (2015)	28.7	5.8	28.9	5.1	37.8	4.7	37.5	5.5
Nascimento IB et al (2020)	28.6	6.2	29.6	6.1	37.5	4.6	37.2	5.7
Syngelaki A et al (2016)	-	-	-	-	-	-	-	-
**Pooled**	**28.3**	**1.0**	**28.8**	**1.7**	**36.4***	**1.2**	**35.7***	**1.5**

From the confidence intervals of the pooled (not shown), the standard error was obtained. And taking as N the number of valid studies included in each pooled, the standard deviation of each pooled was calculated. *Statistically significant differences

C. Maternal weight gain

Reference	Age				BMI			
	Intervention		Control		Intervention		Control	
	$\bar{x}$	SD	$\bar{x}$	SD	$\bar{x}$	SD	$\bar{x}$	SD
**Physical exercise**								
Barakat R et al (2009)-1	-	-	-	-	-	-	-	-
Barakat R et al (2009)-2	-	-	-	-	-	-	-	-
Bisson M et al (2015)	30.5	3.7	31.0	4.0	34.6	5.4	33.9	4.5
Daly N et al (2017)	30.0	5.1	29.4	4.8	34.7	4.6	34.7	5.1
Garnæs KK et al (2016)	31.3	3.8	31.4	4.7	33.9	3.8	35.1	4.6
Kong K et al (2014)-1	26.2	2.6	27.3	3.6	26.5	1.2	27.4	1.4
Kong K et al (2014)-2	28.6	5.3	25.7	4.0	34.7	4.6	34.2	3.6
Nascimento SL et al (2011)	29.7	6.8	30.9	5.9	34.8	6.6	36.4	6.9
Oostdam N et al (2012)	30.8	5.2	30.1	4.5	33.0	3.7	33.9	5.6
Ruiz JR et al (2013)	-	-	-	-	-	-	-	-
Seneviratne SN et al (2016)	-	-	-	-	32.1	4.4	34.1	5.9
Wang C et al (2017)	32.1	4.6	32.5	4.9	26.8	2.7	26.8	2.8
**Pooled**	**30.1**	**1.9**	**29.9**	**1.9**	**32.3***	**3.9**	**32.9**	**4.3**
**Metformin**								
Abd El Fattah EA et al (2016)	26.9	5.2	26.2	5.5	36.4	1.0	36.2	0.9
Chiswick C et al (2015)	28.7	5.8	28.9	5.1	37.8	4.7	37.5	5.5
**Pooled**	**27.8**	**1.3**	**27.6**	**1.9**	**37.1***	**0.9**	**36.8**	**0.9**

From the confidence intervals of the pooled (not shown), the standard error was obtained. And taking as N the number of valid studies included in each pooled, the standard deviation of each pooled was calculated. *Statistically significant differences

Table S7. Results for direct pairwise comparisons and network meta-analysis among pregnant women with obesity.

A. Gestational diabetes mellitus

	**Control**	**Aerobic**	**Combined**	**Resistance**	**Metformin**
**Control**		1.82 (0.41, 8.20)	0.98 (0.71, 1.36)		0.78 (0.59, 1.02)
**Aerobic**	1.91 (0.38, 9.60)		-		-
**Combined**	0.85 (0.49, 1.46)	0.44 (0.08, 2.44)			-
**Resistance**					
**Metformin**	0.75 (0.51, 1.09)	0.39 (0.07, 2.05)	0.88 (0.46, 1.67)		

B. Hypertensive disorders of pregnancy

**Control**		2.41 (0.37, 15.65)	0.62 (0.24, 1.58)		0.48 (0.19, 1.22)
**Aerobic**	2.58 (0.26, 25.62)		-		-
**Combined**	0.68 (0.16, 2.82)	0.26 (0.02, 3.91)			-
**Resistance**					
**Metformin**	0.46 (0.18, 1.13)	0.18 (0.02, 2.10)	0.68 (0.12, 3.72)		

C. Maternal weight gain

**Control**		-1.13 (-3.53, 1.28)	0.04 (-1.08, 1.15)	-1.30 (-4.34, 1.74)	-2.82 (-7.26, 1.62)
**Aerobic**	-1.05 (-4.82, 2.73)		-	-	-
**Combined**	-0.03 (-1.93, 1.88)	1.02 (-3.21, 5.25)		-	-
**Resistance**	-1.30 (-5.82, 3.22)	-0.25 (-6.14, 5.64)	-1.27 (-6.18, 3.63)		-
**Metformin**	-2.88* (-5.37, -0.40)	-1.84 (-6.35, 2.68)	-2.86 (-5.99, 0.27)	-1.58 (-6.74, 3.57)	

Light gray indicates lack of direct and indirect comparisons. Dark gray separates direct from indirect comparisons. Gestational diabetes mellitus and hypertensive disorders of pregnancy were measured as risk ratio (95% CI). Maternal weight gain was measured as mean difference (95% CI). Upper diagonal: standard meta-analysis. Lower diagonal: network meta-analysis estimates. *Statistical significance.

Figure S1. Direct pairwise comparisons by outcome.

A. Gestational diabetes mellitus

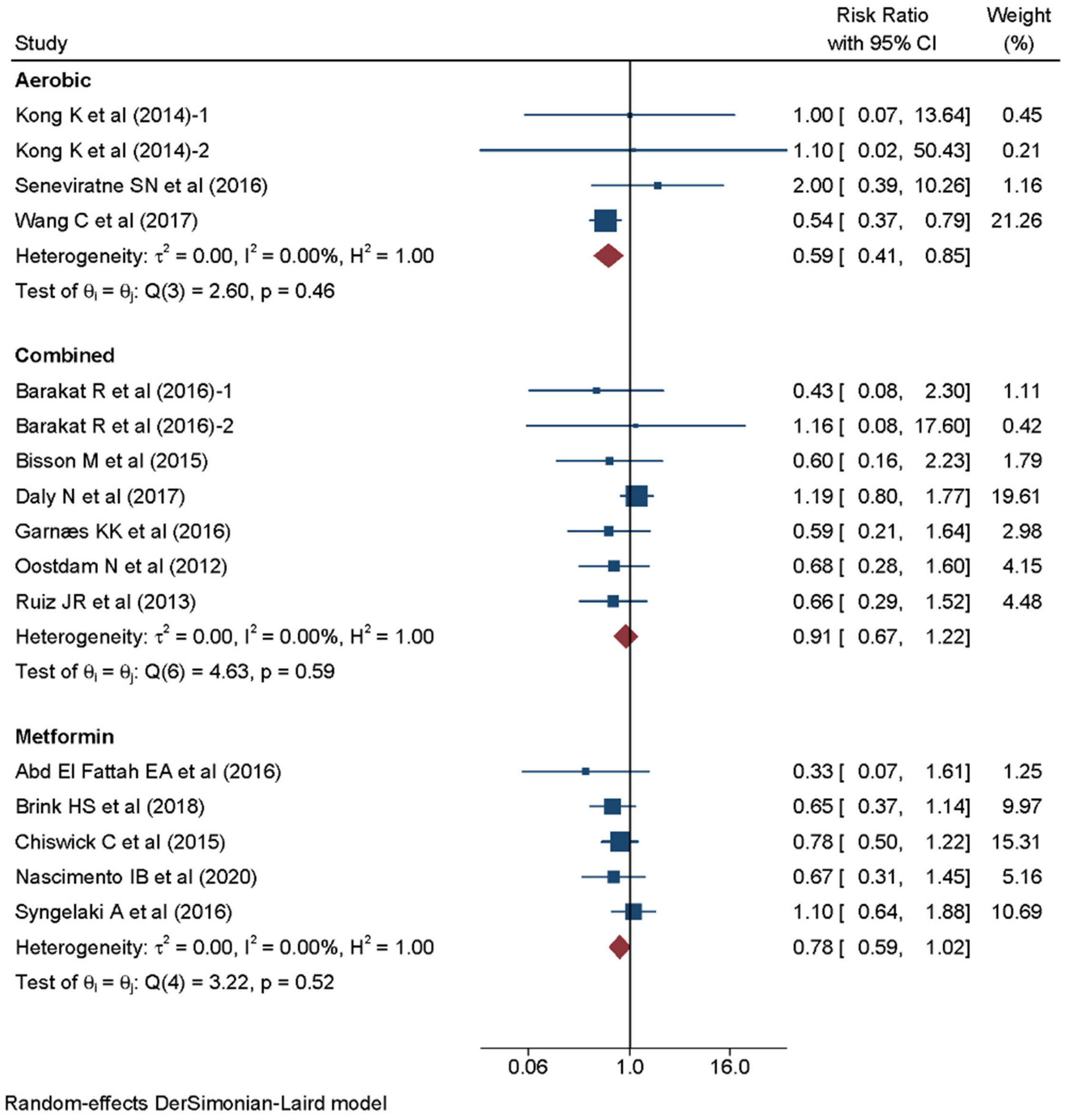

Effect size of individual studies shown in blue (square), and effect size of pooled shown in red (rhombus).

B. Hypertensive disorders of pregnancy

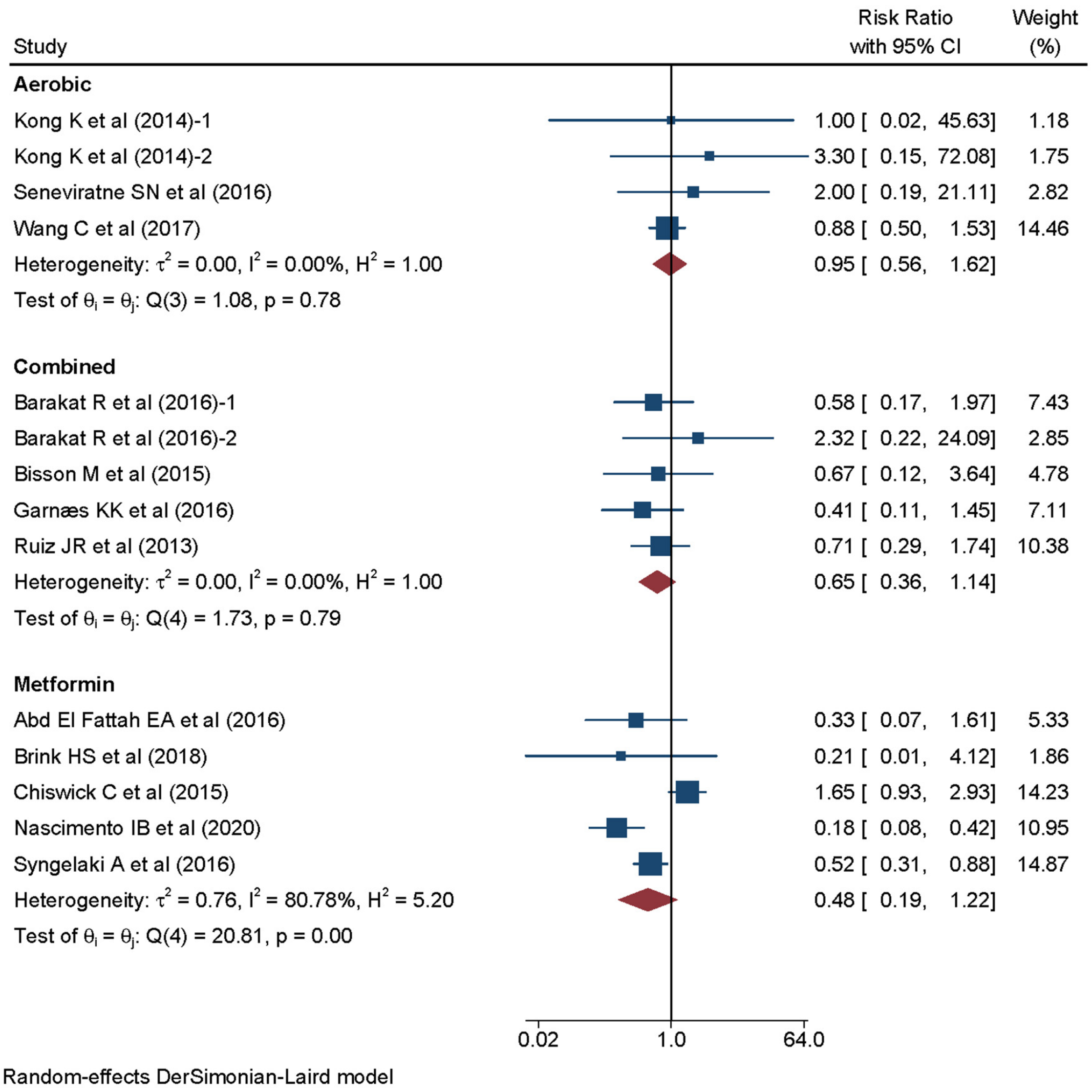

Effect size of individual studies shown in blue (square), and effect size of pooled shown in red (rhombus).

C. Maternal weight gain

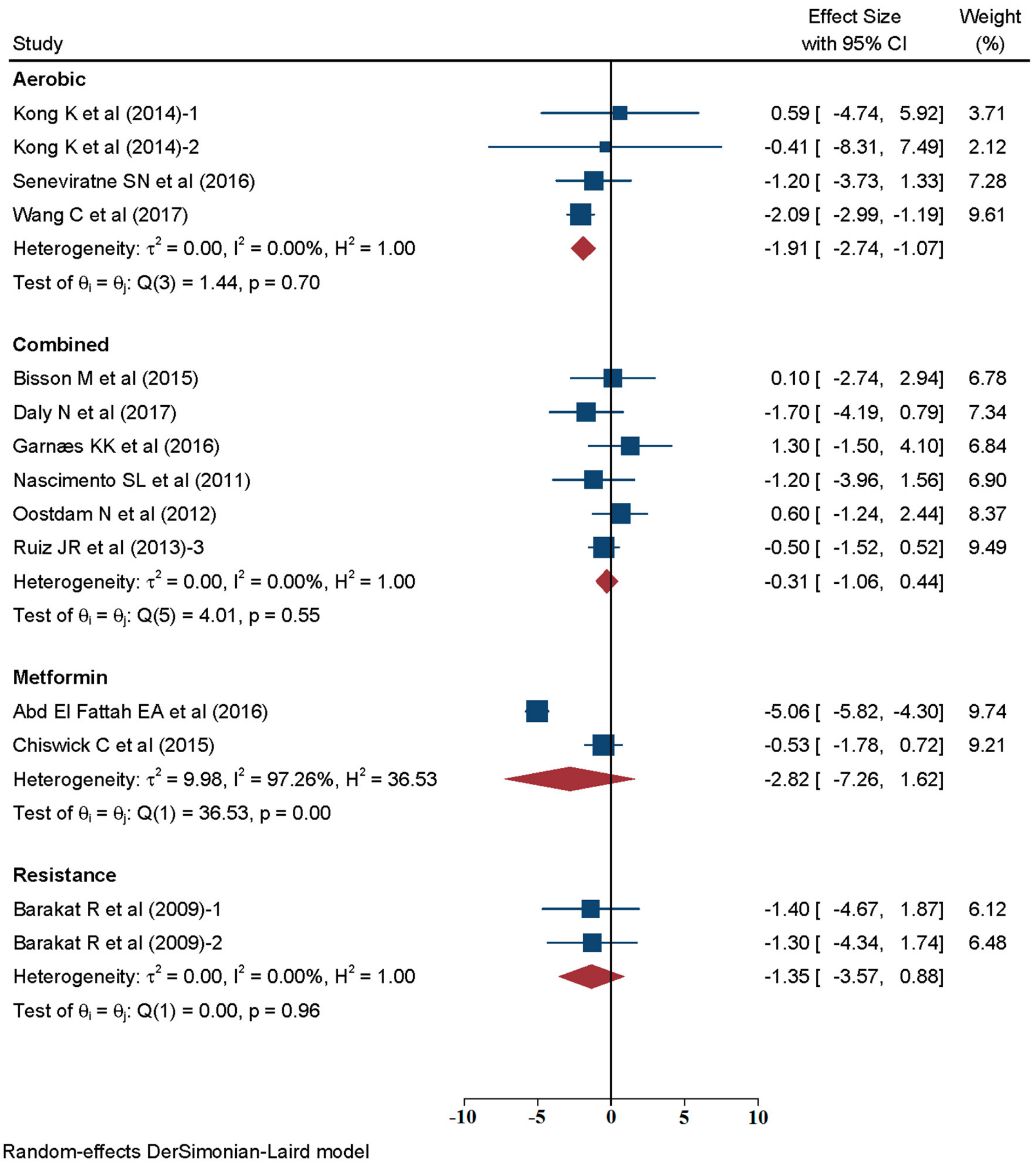

Effect size of individual studies shown in blue (square), and effect size of pooled shown in red (rhombus).

Figure S2. Risk of bias.

A. Risk of bias summary

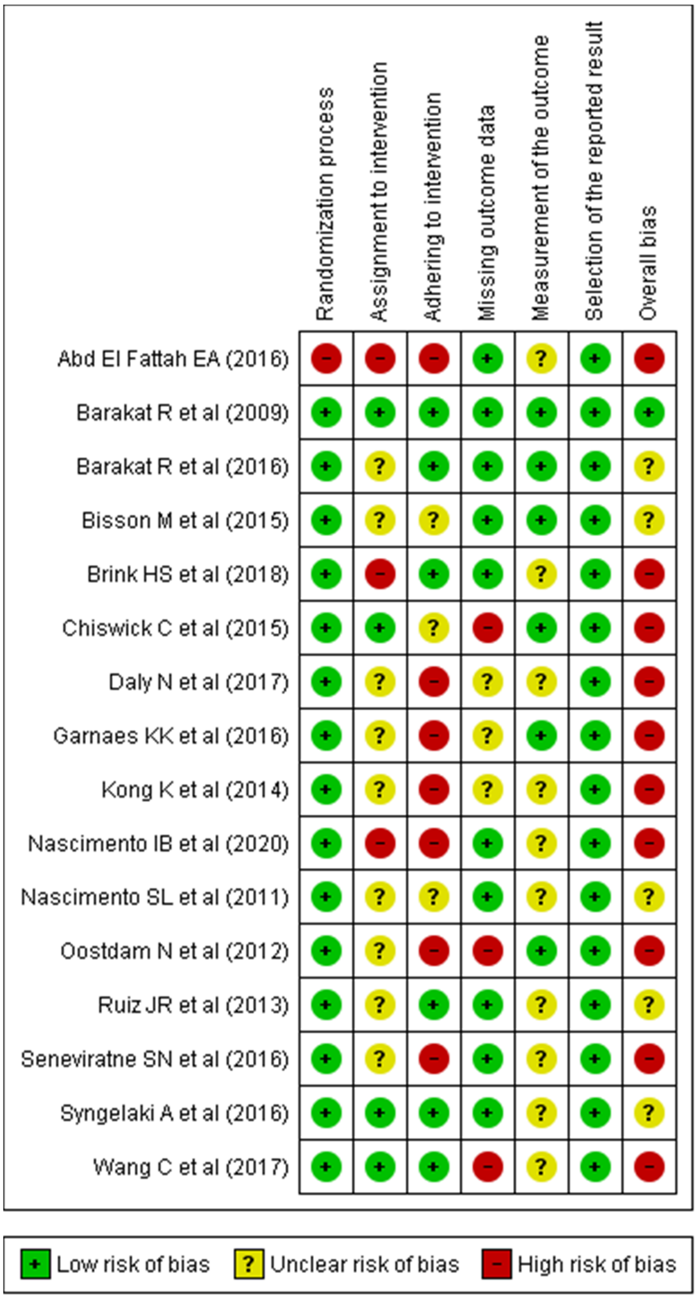

B. Risk of bias graph.

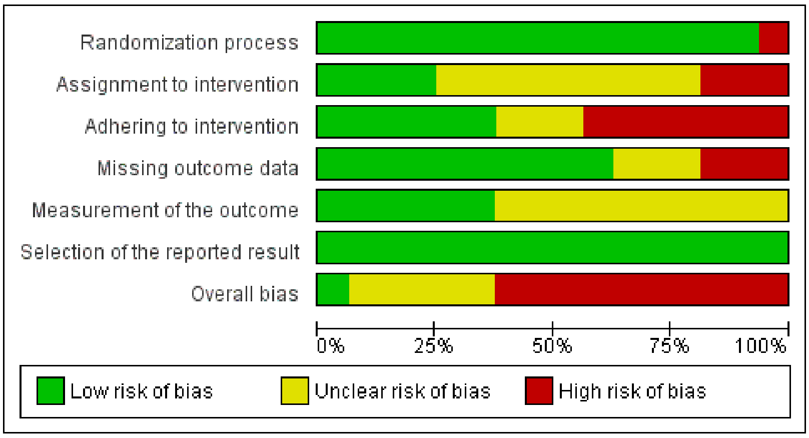

Figure S3. Cumulative probabilities by outcome.

A. Gestational diabetes mellitus

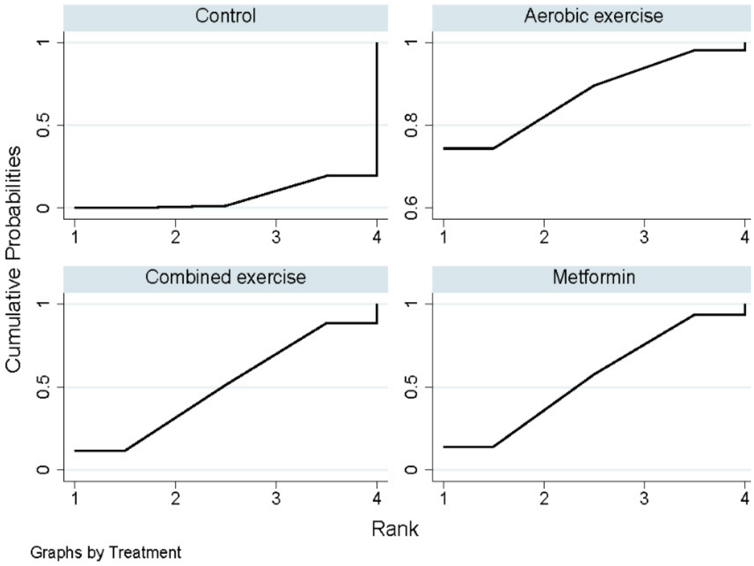

B. Hypertensive disorders of pregnancy

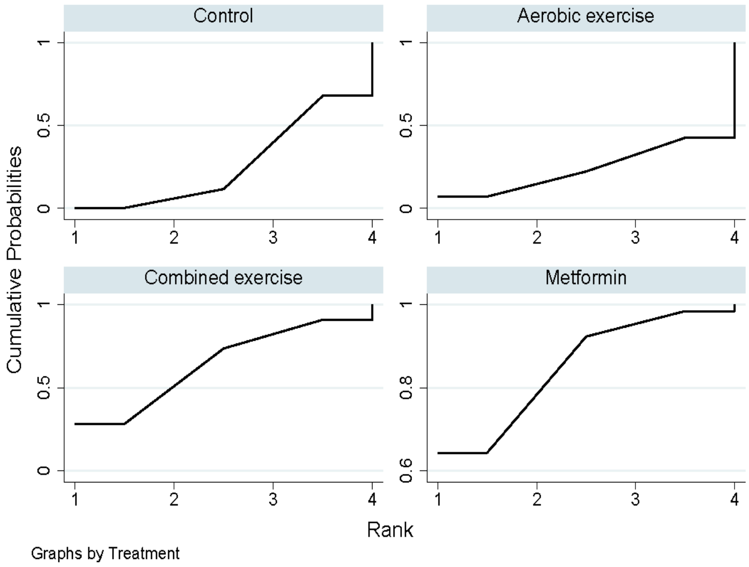

C. Maternal weight gain

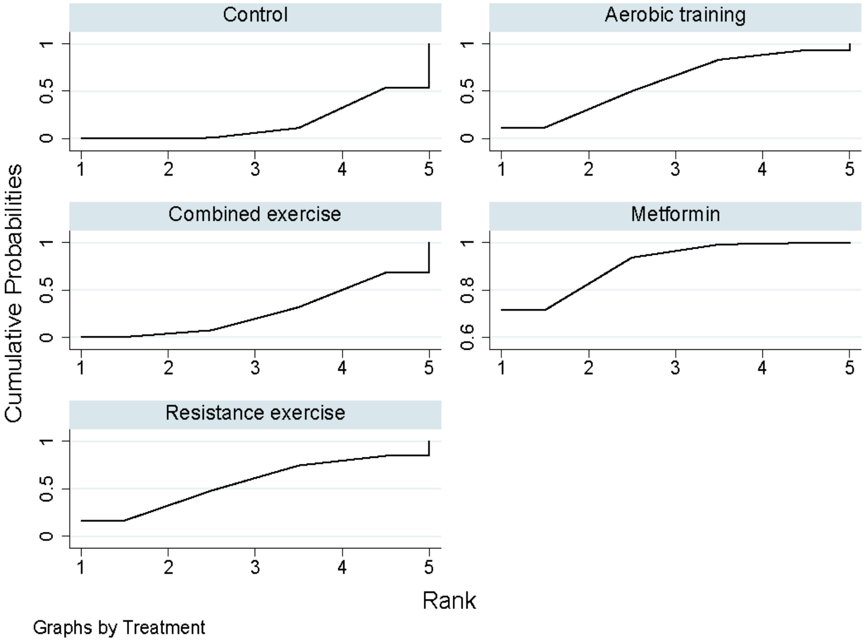

Figure S4. Direct pairwise comparisons and network meta-analysis by outcome among pregnant women with obesity.

A. Gestational diabetes mellitus

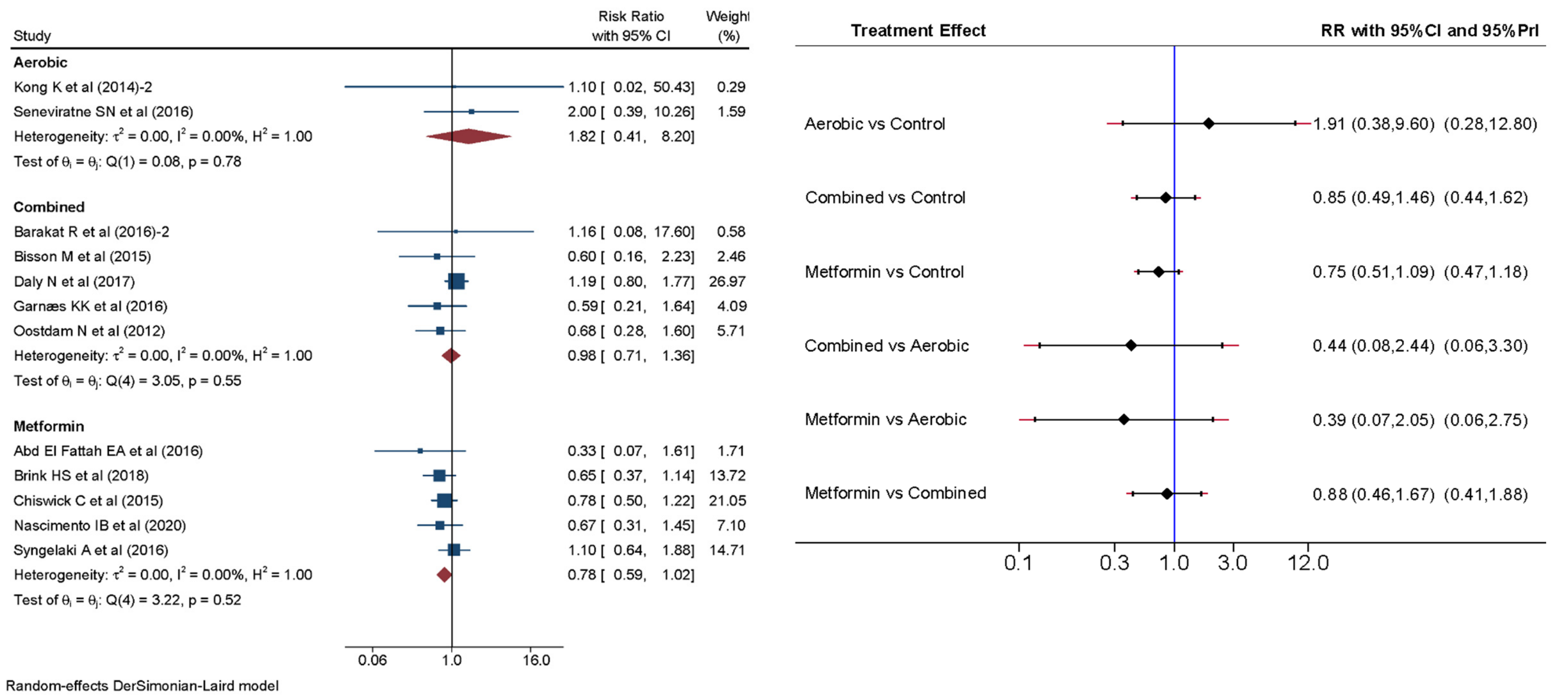

B. Hypertensive disorders of pregnancy

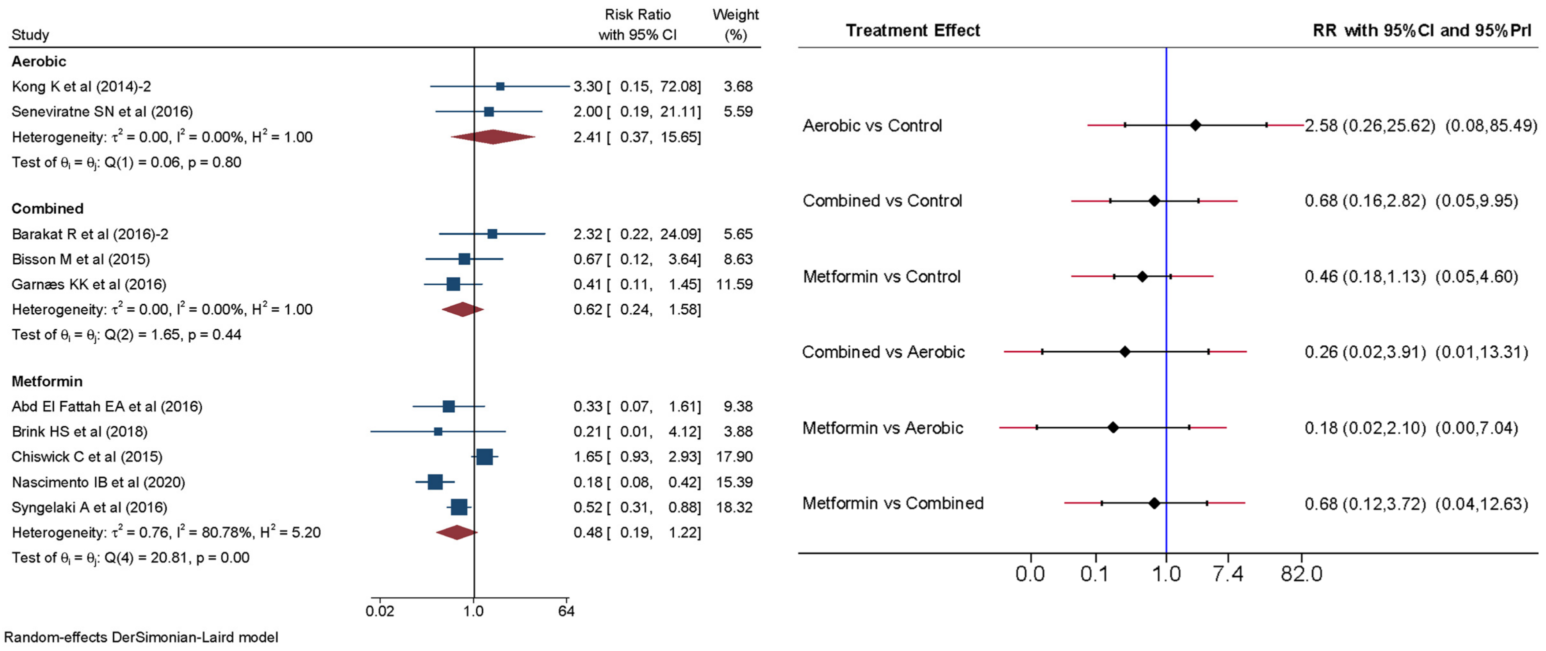

C. Maternal weight gain

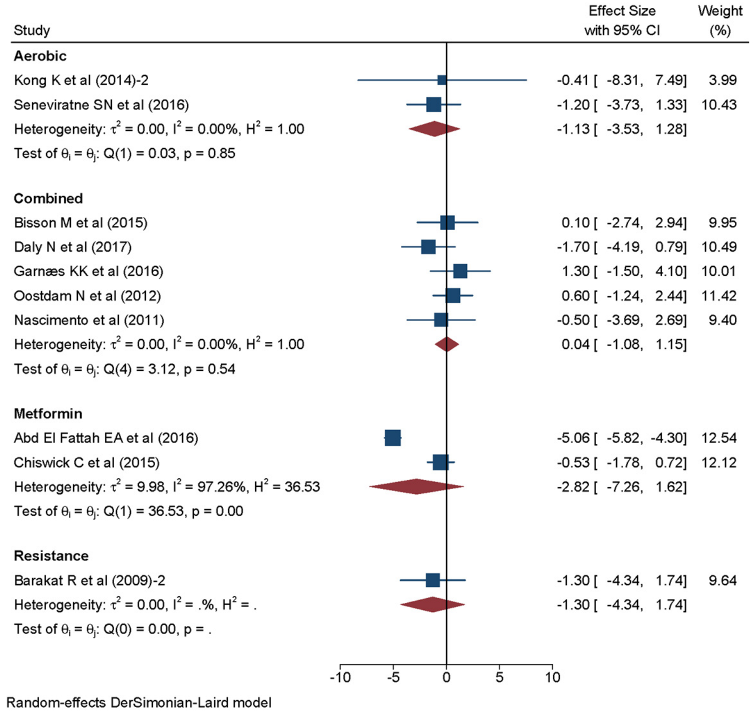

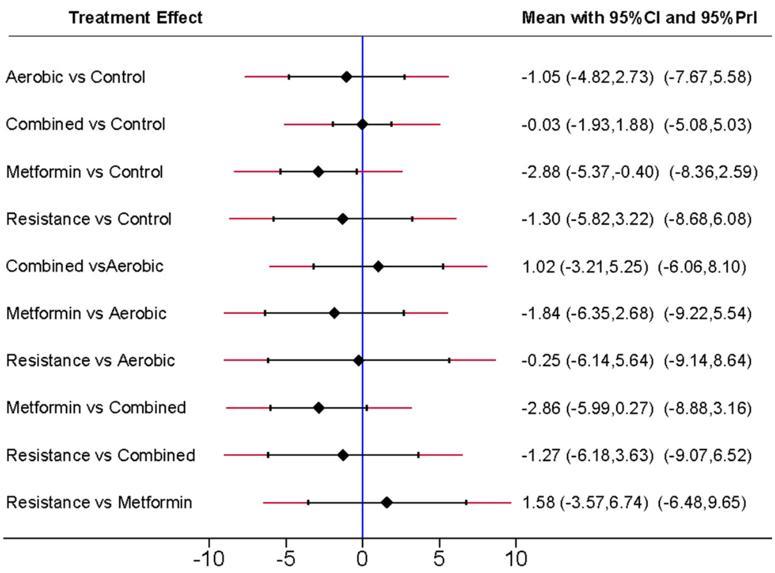

Left, standard meta-analyses; right, network meta-analysis estimates. Risk of GDM and HDP measured as risk ratio (RR, and 95% confidence interval); MWG measured as mean difference (Mean, and 95% confidence interval).

Figure S5. Funnel plot by outcome.

A. Gestational diabetes mellitus

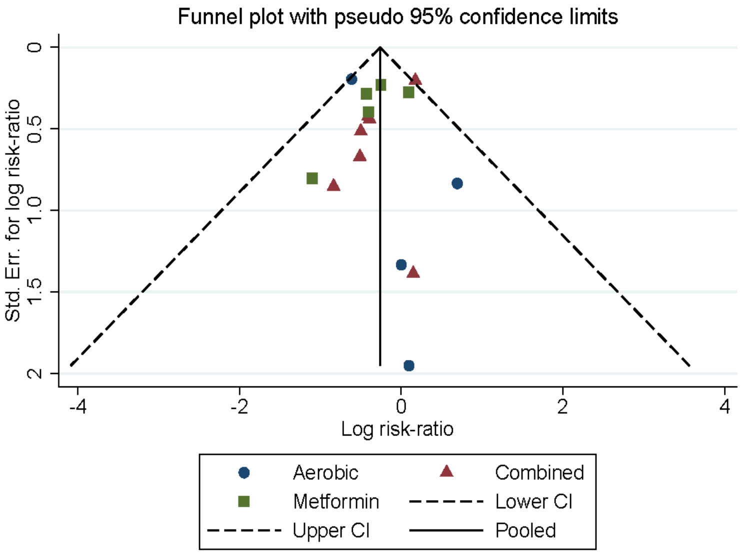

B. Hypertensive disorders of pregnancy

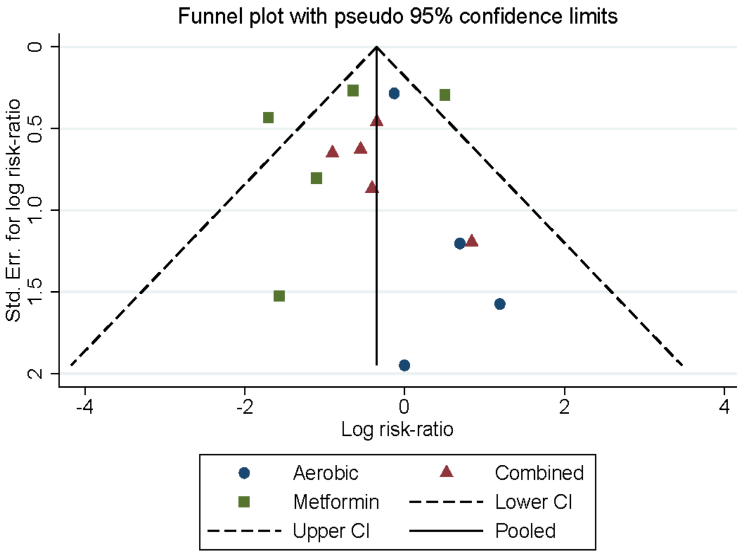

C. Maternal weight gain

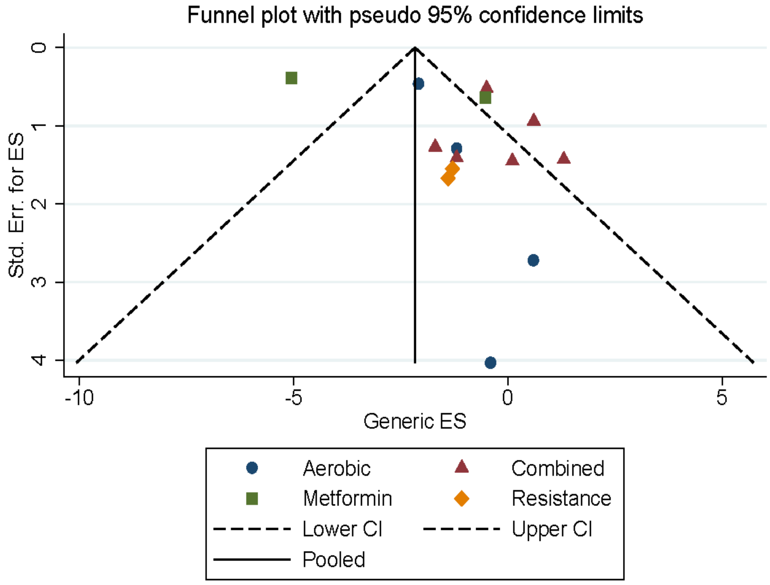

Supplement: Supplementary file 1 [file jcm-10-03490-s001.zip › jcm-1330324-supplementary.pdf]
